# Fractionation and Chemical Characterization of Cell-Bound Biosurfactants Produced by a Novel *Limosilactobacillus fermentum* Strain via Cheese Whey Valorization

**DOI:** 10.3390/foods14244342

**Published:** 2025-12-17

**Authors:** Dimitra Alimpoumpa, Harris Papapostolou, Maria Alexandri, Vasiliki Kachrimanidou, Nikolaos Kopsahelis

**Affiliations:** Department of Food Science and Technology, Ionian University, 28100 Argostoli, Greece; dimialib2014@yahoo.com (D.A.); harris_papapostolou@yahoo.gr (H.P.); malexandri@ionio.gr (M.A.); v.kachrimanidou@ionio.gr (V.K.)

**Keywords:** lactic acid bacteria, biosurfactants, cheese whey valorization, biosurfactant stability, amino acid analysis, structural characterization, column fractionation

## Abstract

Lactic acid bacteria (LAB) have attracted scientific attention as potential producers of biosurfactants (BS); however, there is limited knowledge on the structure of the produced molecules. The aim of this study was to elucidate the individual components comprising the crude BS produced by *Limosilactobacillus fermentum* ACA-DC 0183. Initially, batch fermentations using substrate recycling were employed, leading to the production of 0.76 g/L of crude BS from cheese whey as the sole carbon and nutrient source. The produced BS maintained their properties under various temperatures, pH values, and salinity levels, signifying their potential uses in food applications. Additionally, the structural components were analyzed after hydrolysis. The lipoic part was mainly composed of palmitic acid, oleic acid, and stearic acid, while 17 amino acids were identified as part of the protein moiety of the molecule. Acid hydrolysis of the carbohydrate moiety revealed that this part consisted of glucose, galactose, and glycerol. Partial purification with column chromatography and characterization using FTIR demonstrated the presence of a glycoprotein and a glycolipid as surface-active molecules. Revealing the structure and specific properties of microbially produced BS can expand their utilization in target applications, while their production from renewable sources contributes towards the sustainable production of LAB-based BS.

## 1. Introduction

Microbially produced surfactants (biosurfactants, BS) have emerged as an eco-friendly alternative to synthetic surfactants, owing to their properties, non-toxicity, and biodegradability [1]. BS can be synthesized by a plethora of microbial strains exhibiting exceptional performance at low concentrations across diverse chemical and environmental conditions. These attributes render these compounds excellent candidates, expanding their potential applications [2]. During the last decade, extensive research has been carried out on the production, properties evaluation, and structural analysis of BS synthesized by various bacterial strains belonging to the genera of *Pseudomonas, Acinetobacter*, *and Bacillus*, as well as from yeasts such as *Candida, Yarrowia, Starmerella, and Pseudozyma* [1,3,4,5,6]. However, their broad application, especially in food formulations, is limited due to the fact that most of these strains are pathogenic or opportunistic pathogenic [7]. Lactobacilli have arisen as alternatives not only due to their GRAS (Generally Recognized As Safe) status but also for their ability to produce BS with significant properties, especially in terms of stability, while also presenting antioxidant and antimicrobial activities [8,9,10,11]. The feasibility of producing BS by cultivating lactic acid bacteria (LAB) on renewable resources, such as cheese whey, has already been demonstrated [12,13,14,15].

The unique properties of BS result from their chemical structure, which, especially in the case of LAB, could be a combination of different compounds, including glycolipids, glycoproteins, proteins, lipopeptides, lipoproteins, and phospholipids [16]. The final structure is influenced not only by the specific strains employed but also by other factors such as the carbon and nitrogen source, salts, trace elements, and the growth phase of the strain [17].

The chemical characterization, as well as the separation and purification of LAB BS, are essential steps for their subsequent applications. The structural diversity, differences in solubility, ionic charge, and production methods can significantly impact their recovery and purity [18]. Spectrophotometric analyses like Lowry and the phenol-sulfuric acid method, Fourier-Transform Infrared Spectroscopy (FTIR), and Thin-Layer Chromatography (TLC) are often used for qualitative and quantitative determination of their structural components, while more sophisticated methods such as liquid chromatography–mass spectrometry (LC–MS) and nuclear magnetic resonance (NMR) spectroscopy are employed to fully elucidate their structure [19].

Only a few studies deal with the structural characterization of LAB-derived BS. Recently, Mouafo et al. [20] investigated the structure of cell-bound biosurfactants produced from the LAB strain *Lactobacillus paracasei* subsp. *tolerans* N2 revealing its glycopeptidic nature. Nataraj et al. [21] indicated that strain *L. acidophilus* NCFM produced BS composed of a mixture of carbohydrates, lipids, and proteins, whereas strain *Lacticaseibacillus rhamnosus* GG synthesized a molecule consisting of proteins and carbohydrates. BS derived from the strain *Limosilactobacillus fermentum* ACA-DC 0183 were characterized as glycolipoproteins by spectrophotometric methods and TLC analysis [12]. A glycolipoprotein was also reported in the work of Lara et al. [2], who studied the BS synthesized by *Lactiplantibacillus plantarum* Tw226, but the researchers did not carry out any additional analyses. A complete molecule characterization has been presented by Saravanakumari and Mani [22]. The authors characterized the BS derived from *Lactococcus lactis* as a xylolipid composed of methyl-2-*O*-β-D-xylopyranoside and octadecanoic acid. Biosurfactants derived from *Lactobacillus plantarum* IRL 560 were chemically characterized as three different glycolipids (GL-1, GL-2, and GL-3) [23]. Glucose and galactose comprised the carbohydrate part of the molecule, which were differently linked depending on the molecule. Individual fatty acid analysis also revealed differences among the three molecules; however, C18:1 n-9 was the predominant fatty acid in all cases. It is evident that the structure of the biotechnologically synthesized BS is strain-dependent. The component characterization is therefore crucial for the elucidation of the chemical structure of the microbially derived BS.

In view of the above, this study focused on functional properties analyses and structural characterization of cell-bound (CB) BS produced by the LAB strain *Limosilactobacillus fermentum* ACA-DC 0183. The strain was initially cultivated in repeated batch fermentation mode using cheese whey (CW) in order to synthesize BS. Subsequently, the crude BS was structurally characterized using high-performance liquid chromatography with diode-array detection (HPLC-DAD), Fourier-Transform Infrared Spectroscopy (FTIR), Thin-Layer Chromatography (TLC), and Gas Chromatography (GC). Partial purification of the crude BS was also employed by means of column chromatography. Taking into account the complexity of these compounds and the scarce studies on their structure, this work paves the way towards a better understanding of the surface-active molecules produced by LAB. Revealing their structure—and the subsequent properties of each individual compound—could disclose new or specific target applications, expanding their market potential.

## 2. Materials and Methods

### 2.1. Microorganism and Culture Conditions

The lactic acid bacterial strain *Limosilactobacillus fermentum*, ACA-DC 0183, was provided by the culture collection of the Laboratory of Dairy Research of Agricultural University of Athens, Greece (ACA-DC). This strain has previously been tested for its ability to produce biosurfactants [12]. The bacterial culture was stored in cryovials containing De Man, Rogosa, and Sharpe (MRS) broth (Condalab, Madrid, Spain) supplemented with 20% (*v*/*v*) glycerol, which were previously autoclaved (121 °C, 15 min). Before use, the strain was streaked on MRS agar plates and incubated at 37 °C for 48 h. Then, a single colony was used to prepare a preculture in shake flasks containing MRS broth, which were incubated at 37 °C for 14–16 h.

### 2.2. Cheese Whey Utilization for Biosurfactant Production

The cheese whey (CW) employed as a substrate for this study was obtained after the production of mizithra cheese and was kindly provided by the “Galiatsatos” dairy industry in Kefalonia. Prior to its use in fermentation, CW was subjected to deproteinization as previously described [24,25]. Briefly, after pH adjustment to 10 with the addition of 5 M NaOH, CW was heated to 55–60 °C for 3 min. Subsequently, the pH value was adjusted to 3.5 for 5 min (HCl 5 M) and then to 4.7 (NaOH 5 M). Finally, CW was autoclaved to precipitate any remaining proteins and then filtered through Whatman paper (Cytiva, Wilmington, NC, USA) (0.45 μm).

### 2.3. Biosurfactant Production Using CW

The fermentation process was carried out in a 2 L bioreactor with a working volume of 1.5 L, with 10% inoculum size. The temperature was maintained at 37 °C, and the pH value was adjusted to 6.7–6.9 using 5 M NaOH. The bioreactor was connected with a controller (SC200, HACH, Loveland, CO, USA) for monitoring temperature and pH throughout the process, while the agitation was set at 300 rpm.

After 4 h of fermentation, the substrate was aseptically separated from the cell biomass by centrifugation (4000 rpm, 15 min, 4 °C, Rotina 420R, Hettich, Kirchlengern, Germany). The bioreactor was then refilled with the cell-free substrate and a fresh 16 h inoculum. The process was repeated three times. During the fermentation, samples were collected to monitor sugar and nitrogen consumption as well as microbial growth and biosurfactant production.

### 2.4. Extraction of Biosurfactants

Cell-bound BS (CB-BS), which are associated with the cell surface of the microorganism, were extracted using phosphate-buffered saline solution (PBS, pH = 7). Bioreactor samples were initially centrifuged (4000 rpm, 15 min, 4 °C, Rotina 420R, Hettich, Germany), and the cell-free supernatant was rejected. The cell pellets were washed twice with demineralized water (dH_2_O) and subsequently resuspended in PBS at a ratio of 1:6 (*v*/*v*) fermentation broth–PBS. The mixture was left for 12 h at 4 °C under continuous agitation following centrifugation to obtain the PBS extracts of crude BS. Afterwards, the extracts were dialyzed using dialysis membranes (6000–8000 Da) in dH_2_O. Subsequently, the dialyzed samples were filtered through Whatman^®^ 0.2 μm filters. The presence of BS was evaluated by measuring the surface tension (ST) of the extracts.

### 2.5. Fractionation of Biosurfactants with Column Chromatography

Separation of the crude cell-bound BS into fractions was carried out using silica gel (60–120 mesh, HiMEDIA, Kennett Square, PA, USA) as the stationary phase, while two different organic solvent mixtures (trial 1 and trial 2) were employed as mobile phases. For each trial, 5 g of silica gel was mixed with 5 mL of chloroform, heated at 60 °C for 10 s to produce a stable phase slurry, and added into the column. The slurry was then washed with 10 mL of chloroform and left for 20 min for further packing. For sample preparation, 0.5 g of crude BS was suspended in 5 mL of ultrapure water and then loaded onto the glass column.

For the first trial, a gradient elution system of increasing polarity was used as the mobile phase, consisting of 50 mL mixtures of chloroform and methanol in the following ratios: 50:0, 50:5, 50:50, and 5:50, following a methodology described previously [26,27,28].

In the second trial, a different solvent system was used, consisting of acetonitrile–H_2_O in a gradient system at the following ratios: 5:95, 20:80, 50:50, 80:20, and 95:5. A total volume of 50 mL was used for each mixture, and fractions of 50 mL were collected. Finally, solvents were evaporated using a rotary evaporator, and the dried fractions were used for further analysis.

### 2.6. Surface Tension Measurements

The surface tension of the extracted crude BS was measured using the Wilhelmy plate method at 25 °C with a K20 Krüss Tensiometer (KRÜSS GmbH, Hamburg, Germany) featuring a 1.9 cm platinum plate (wetted length: 40.20 mm) [29]. Prior to every measurement, the tensiometer was calibrated with distilled water. BS production was evaluated by comparing the reduction in surface tension against the control sample (distilled water and PBS). Each measurement was performed in triplicate (n = 3), and the results are presented as mean values. The presence of BS was evaluated throughout the surface tension measurement.

### 2.7. Stability Tests

Stability tests were conducted on crude CB-BS solutions at the critical micelle concentration (CMC, 0.6 g/L), testing different temperatures, pH values, and degrees of salinity. The stability against various temperatures was evaluated by subjecting samples to different temperatures ranging from 4 °C to 90 °C for one hour, as well as at 121 °C for 20 min. The effect of pH was examined by fixing the desirable pH value (from 2 to 12), while salinity was evaluated at solutions with 1.5 g/L and 9 g/L NaCl. Surface tension was measured before and after each condition to evaluate the stability of the crude CB-BS [10,30].

### 2.8. Analytical Methods

#### 2.8.1. Lactose, Lactic Acid, and Monosaccharides Determination

Lactose consumption and lactic acid production during CW fermentation were determined by means of HPLC using an Agilent 1200 series (Agilent, Santa Clara, CA, USA) equipped with an ROA-organic acid H+ column (300 mm × 7.8 mm, Phenomenex, Torrance, CA, USA) coupled to a Refractive Index Detector (RID). The mobile phase was 10 mM H_2_SO_4_ with a flow rate of 0.6 mL/min, the column temperature was set to 65 °C, and the injection volume was 10 μL. Prior to analysis, the samples were appropriately diluted and filtered through Whatman^®^ (0.2 μm).

#### 2.8.2. Analysis of Total Dry Weight (TDW)

Total dry weight (TDW, g/L) of the microbial biomass was measured gravimetrically. Briefly, an adequate amount of fermentation broth was centrifuged (Nuve NF 048, Nüve, Ankara, Turkey), and the microbial cells were washed with dH_2_O to remove any substrate residues. Then the microbial pellet was dried at 65 °C until constant weight.

#### 2.8.3. Spectrophotometric Methods

Free amino nitrogen (FAN) consumption was estimated using the ninhydrin method [31]. Protein content of CB-BS was estimated by the Lowry method [32] and carbohydrate content using the Dubois protocol [33].

#### 2.8.4. Total Lipid Content Determination

Total lipid content was determined based on the protocol followed by Patel et al. [34]. More specifically, 0.5 g of dry crude BS was resuspended in 20 mL of chloroform–methanol (2:1 *v*/*v*) mixture and left in the dark for 24 h. Subsequently, the sample was centrifuged, and the supernatant was filtered using Whatman filter paper with a pore size of 0.45 μm. Five milliliters of 0.075% MgCl_2_·6H_2_O were then added, followed by centrifugation at 4000 rpm for 5 min. The organic phase was collected and washed three times with 1 mL of KCl (2 N): methanol (4:1 *v*/*v*) to ensure further purification. Finally, the solvents were evaporated, and the total lipid content was determined gravimetrically, expressed as grams of lipids per gram of dry crude BS.

#### 2.8.5. Structural Analysis of BS Using FTIR

The identification of the functional groups present in crude BS and purified BS fractions was carried out with an Agilent Cary 630 FTIR spectrometer equipped with an ATR sampling module with a diamond crystal. The analysis was conducted in a range of 4000–400 cm^−1^ with a resolution of 4 cm^−1^.

#### 2.8.6. Carbohydrate Analysis of Biosurfactants

To identify the individual monosaccharides present in the carbohydrate moiety of the BS, the samples were initially hydrolyzed and then analyzed with HPLC. Hydrolysis was carried out in a screw-capped test tube containing 60 mg of BS mixed with 25 mL of dH_2_O. Subsequently, 0.1 mL of trifluoroacetic acid was added, and the samples were autoclaved at 121 °C for 2 h to achieve carbohydrate hydrolysis. The samples were allowed to reach room temperature, and then the pH was adjusted to 7 using a 25% ammonia solution. Monosaccharide analysis was performed as described in Section 2.8.1.

#### 2.8.7. Amino Acid Analysis of Biosurfactants

##### Extraction of Free Amino Acids

Extraction of free amino acids present in the crude BS was carried out following the protocol of Kowalska et al. [35]. Briefly, 0.5 g of crude BS was mixed with 2 mL of TCA (1% *w*/*v*) and 0.5 mL of 70% ethanol for 60 min under continuous agitation. Then the sample was centrifuged at 9000 rpm for 15 min, and the supernatant was collected and filtered. The filtrate was transferred into a 25 mL volumetric flask, and the volume was made up with distilled water. The amount of free amino acids was then determined by RP-HPLC.

##### Hydrolysis of the Peptide Fraction of BS

The protein fraction of the produced BS was hydrolyzed prior to amino acid analysis based on the protocol described by Shen et al. [36]. More specifically, 100 mg of crude BS were mixed with 10 mL of 6 N HCl, containing 0.15 mL of phenol. The samples were placed at −80 °C for 30 min and then incubated at 105 °C for 24 h. Afterwards, the samples were left at room temperature to cool down, and diluted to 50 mL with dH_2_O. A portion of 4 mL of the hydrolyzed sample was freeze-dried and subsequently suspended in 2 mL of citric acid buffer and vortexed for 10 s.

##### RP-HPLC Analysis of Amino Acids

The analysis of both free amino acids and the hydrolyzed protein fraction of the biosurfactants was performed using high-performance liquid chromatography (HPLC, Agilent, Santa Clara, CA, USA) equipped with a diode-array detector (DAD) and a Zorbax Eclipse Plus C18 column (4.6 × 150 mm, 5 μm particle size, Agilent, Santa Clara, CA, USA). The method followed the Agilent protocol for amino acid analysis as described by Natsia et al. [37]. More specifically, the mobile phase consisted of 10 mM Na_2_HPO_4_:10 mM Na_2_B_4_O_7_:5 mM NaN_3_ (Solvent A) and acetonitrile–methanol–water (45:45:10, *v*:*v*:*v*) (Solvent B), at a flow rate of 1 mL/min. The gradient elution program was as follows: 0–0.84 min, 98% Solvent A and 2% Solvent B; at 33.4 min, 43% Solvent A and 57% Solvent B; at 33.5 min, 100% Solvent B; 33.5–39.3 min, 100% Solvent B; at 39.4 min, 98% Solvent A and 2% Solvent B; and 39.4–60 min, 98% Solvent A and 2% Solvent B. Column temperature was set at 40 °C. Sample derivatization was carried out automatically in the autosampler prior to injection (pre-column derivatization), using ortho-phthalaldehyde (OPA) and fluorenylmethoxy chloroformate (FMOC) reagents. OPA was prepared by diluting 10 mg in 1 mL borate buffer (0.4 M, pH 10.2) with 23 μL of 3-mercaptopropionic acid. For FMOC, 2.5 mg were diluted in 1 mL acetonitrile. The chromatograms were monitored at 262 and 338 nm.

#### 2.8.8. Determination of Fatty Acid Methyl Esters with Gas Chromatography (GC)

The compositional analysis of the lipoic fraction was carried out using Gas Chromatography (SHIMADZU GC2010 Pro, Shimadzu, Kyoto, Japan) operating with a Flame Ionization Detector (FID) after transesterification of fatty acids, following the protocol of Patel et al. [34]. More specifically, samples were methyl esterified by dissolving 4–6 mg in 1 mL of methanolic boron trifluoride (BF_3_) solution, followed by heating at 80 °C for 20 min in a water bath. The methyl esters were then extracted by adding 1 mL of deionized water and 2 mL of hexane. The upper phase (hexane phase) was collected and dried using anhydrous sodium sulfate. The sample injection was conducted using a split-splitless injector in split mode with a split ratio of 1:100 using a MEGA-10 column (30 m × 0.25 mm, film thickness 0.25 μm). Helium was used as the mobile phase carrier gas at a flow rate of 3 mL/min. The temperature of the column was initially set at 60 °C for one minute, then increased by 8 °C/min until 245 °C, where it was held for 5 min. The identification of specific fatty acids was carried out using a standard FAME mixture (Supelco 37 Component FAME mix, Merck, Sigma-Aldrich, Darmstadt, Germany) and expressed as area percentage.

### 2.9. Statistical Analysis

Statistical analysis tests were applied to evaluate significant differences at a level of 5% (*p* < 0.05) among treatments via analysis of variance (ANOVA) and Tukey’s HSD (honest significant difference). The tests were carried out using the Microsoft Excel software (version 2010).

## 3. Results and Discussion

### 3.1. Biosurfactants Production in Repeated Batch Fermentation with Substrate “Recycling”

**Figure 1:**
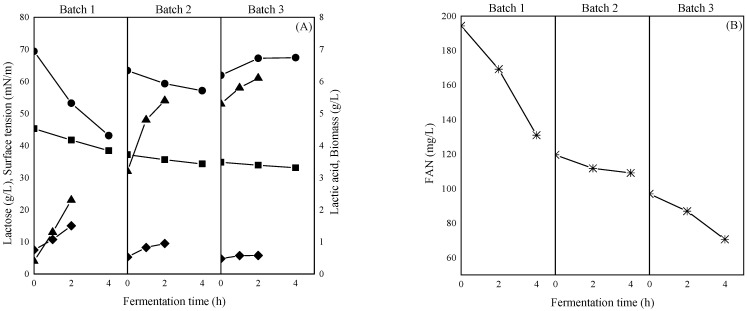
BS production in repeated batch mode using substrate recycling. (**A**) Lactose consumption (■), lactic acid production (▲), total cell dry weight (TDW) (♦), and ST reduction (●); (**B**) FAN consumption.

In our previous work [12], strain ACA-DC 0183 had the ability to produce BS at higher concentrations during the first 4 h of fermentation, as evidenced by the abrupt drop in the ST. To this end, a repeated batch strategy, with substrate “recycling” was tested, aiming to fully exploit the lactose present in cheese whey towards the highest possible BS production. The medium was solely composed of cheese whey, without any external nutrient supplementation. Fermentation results are presented in Figure 1. The first batch lasted 4 h, during which almost 7 g/L of lactose were consumed. At the same time, lactic acid was produced (1.9 g/L), while ST was reduced from 69.4 mN/m to 43.1 mN/m from the formation of 0.36 g/L crude BS. Lactose consumption dropped to 2.9 and 1.7 g/L for the 2nd and 3rd batches (Figure 1A). A decrease in ST was also observed, however, at lower rates compared to the 1st batch, as the production of BS was 0.3 g/L and 0.1 g/L, respectively. This strategy led to a total production of 0.76 g/L crude BS, a 38% increase when compared to the batch fermentation presented by Kachrimanidou et al. [12].

The decline in the fermentation performance was evident in the 2nd and 3rd batches, even though high concentrations of lactose were still unconsumed (37 g/L and 34 g/L, respectively). Therefore, it can be postulated that the critical factor affecting growth performance is the absence of other essential nutrients, such as essential amino acids, vitamins, or minerals. LAB are characterized as fastidious bacteria, exhibiting certain nutritional requirements, as already demonstrated in many other studies [38,39,40,41]. As shown in Figure 1B, the initial FAN concentration in the 1st batch was 194.3 mg/L, and the strain consumed 63 mg/L. FAN concentration was 133 mg/L and 114 mg/L at the beginning of the 2nd and 3rd batches, while the consumption ceased compared to the 1st batch, with values at 19 and 21 mg/L, respectively. Nutrient requirements greatly differ among the various LAB, with some strains showing explicit preference for free amino acids [38,39], others for low molecular weight peptides [42], while others adopt their consumption requirements based on the abundance of specific amino acids in the medium [38]. Vallejo et al. [43] corroborated the need to use complex nitrogen sources, such as yeast extract, meat extract, and peptone, for the efficient BS production from *Lactobacillus plantarum* ATCC 8014, *Lactobacillus acidophilus* NRRL B 4495, and a *Lactobacillus* sp. strain. Meng et al. [40] demonstrated that the supplementation of milk with asparagine, aspartic acid, cysteine, leucine, methionine, riboflavin, guanine, uracil, and magnesium ions favored cell viability of *L. acidophilus* LA-5. Therefore, it can be deduced that the lack of essential amino acids could affect biomass production (Figure 1A), resulting in lower amounts of lactic acid and BS, as both metabolites are directly correlated to cell proliferation. Nevertheless, a comprehensive study regarding the specific nutritional requirements of strain ACA-DC 0183 should be carried out in order to elucidate the effect of certain compounds on cell growth and BS synthesis.

The repeated batch strategy with substrate recycling enhances BS production and represents a promising route towards the efficient valorization of cheese whey. Future studies should focus on nutrient supplementation in order to achieve complete lactose consumption and higher BS production. For instance, Olszewska–Widdrat et al. [38] evaluated different yeast extract concentrations on lactic acid production and growth of a *Bacillus coagulans* strain. Their findings also presented different consumption patterns of essential amino acids from the bacterium, depending on the initial yeast extract concentration. In another study, Souza et al. [44] evaluated the effect of pH on BS, lactic acid, and bacteriocin production from strain *Lactococcus lactis* subsp. *lactis* CECT-4434. BS synthesis was favored in the absence of pH control, in contrast to lactic acid production, which required pH values of 5.0–5.3. These strategies could help mitigate the issue of low lactose consumption from strain ACA-DC 0183 and improve its performance.

The crude BS that were collected were then subjected to stability evaluation and then to individual component analysis to reveal their structure.

### 3.2. Evaluation of BS Stability 

**Figure 2:**
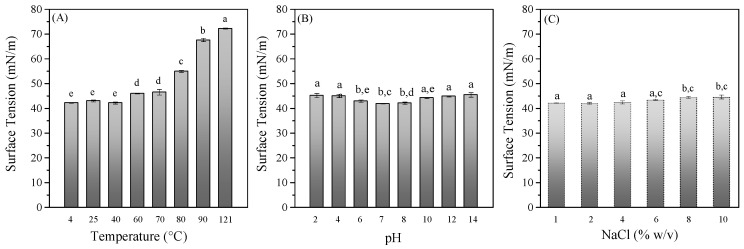
Stability study of BS derived from *L. fermentum* ACA-DC 0183 at various (**A**) temperatures, (**B**) pH values, and (**C**) salinity concentrations. Results are presented as mean ± standard deviation from triplicate experiments of surface tension measurements. Different lowercase letters present significant differences (*p* < 0.05) among the variables.

The tolerance of BS to temperature, pH, and salinity is a critical factor influencing their potential applications in various fields, especially in food systems. The stability of the crude BS derived from the *L. fermentum* ACA-DC 0183 strain to different temperature conditions was assessed by subjecting the samples to a series of temperatures: 4 °C, 25 °C, 40 °C, 60 °C, 70 °C, 80 °C, 90 °C, and 121 °C (Figure 2A). The results indicated that temperatures above 60°C affected the ability of the crude BS to reduce the surface tension. Temperatures up to 70 °C for 1 h led to a slight increase in the surface tension measurements from 42.2 to 46.5 mN/m. As the temperature increased to 80 °C, the surface tension of the sample was increased, reaching a value of 55 mN/m. On the other hand, temperatures above 90 °C were detrimental, as surface tension increased above 67 mN/m (Figure 2A).

Moreover, the crude BS exhibited considerable tolerance over a wide range of pH values (Figure 2B). The results demonstrated that the BS were capable of partially maintaining their stability in both acidic and alkaline environments, as they effectively reduced the surface tension of water across a pH range of 2, 4, 6, 7, 8, 10, and 12, with values ranging between 41.8 and 45.4 mN/m. The most favorable pH values were around 6 to 8, while at extreme acidic or alkaline conditions, a slight increase in surface tension measurements was observed, which may be attributed to the denaturation of the peptide part of the molecule. The effect of acidic conditions was also reported in the studies of Ghasemi et al. [45] and Gudiña et al. [46]. Additionally, the instability of BS under acidic conditions arises from the protonation of negatively charged groups situated at the polar ends of the molecules [47].

Salinity (in the range of 1–10% NaCl) had little effect on the ability of the crude BS to reduce surface tension (Figure 2C). These results are in line with other studies on lactobacilli-derived BS. For example, Behzadnia et al. [10] also observed no effect of NaCl concentrations up to 10% on BS produced by a *Lactobacillus plantarum* strain, while Sakr et al. [30] tested concentrations up to 14%, which did not significantly influence the emulsifying activity of a glycolipopeptide type of BS derived from another *L. plantarum* strain.

### 3.3. Structural Characterization of the Crude Biosurfactants

According to the findings of Kachrimanidou et al. [12], the crude BS produced from *L. fermentum* ACA-DC 0183 could be characterized as a glycolipopeptide, based on preliminary TLC analyses and spectrophotometric results. To this end, the analysis of the individual components of each moiety (lipoic, proteinaceous, carbohydrate) was carried out in order to clarify the molecule’s composition.

#### 3.3.1. Fatty Acid Analysis

A total of 20 fatty acids were identified from the GC-FID analysis of the lipoic fraction derived from the crude BS (Table 1). The analysis revealed that palmitic acid (C16:0) was the most abundant fatty acid, corresponding to 27.9% of total peaks, followed by oleic acid (18.7%) and stearic acid (11.22%). Palmitic acid has been identified as the primary fatty acid in LAB-derived BS by many researchers [20,26,48,49,50,51]. Myristic and decanoic acids were also found in lower amounts (4.3% and 2.2%), while lauric acid, linoleic, palmitoleic, and henicosanoic acid altogether comprised 5% of total fatty acids in the fraction (Table 1). The lipoic composition can vary greatly depending on the strain and culture conditions. The glycolipopeptide produced by *L. plantarum* 1625 was composed of long-chain fatty acids, primarily from palmitic acid, along with octadecadienoic acid, methyl stearate, and cyclononasiloxane [50]. Al-Janabi et al. [51] reported palmitic acid as one of the main fatty acids comprising a glycolipopeptide from another *L. plantarum* strain. On the other hand, cultivation of strain *L. plantarum* JBC5 on ghee (clarified butter) enriched with MRS medium led to the production of a cyclic lipopeptide, similar to surfactin [52]. Oleic acid was the predominant fatty acid composing the glycolipids identified from Sauvageau et al. [23], whereas the xylolipid reported by Saravanakumari & Mani [22] contained only octadecanoic acid. Nataraj et al. [21] identified the presence of hexadecanoic acid, 9-octadecenoic acid, and methyl stearate in the lipoic part of *L. acidophilus* NCFM biosurfactants. The purified mannosylerythritol lipids (MEL) obtained by Andrade et al. [53] contained exclusively shorter chain fatty acids (C8:0, C10:0, C12:1, C12:0, C14:0, and C18:1).

#### 3.3.2. Amino Acid Analysis

TLC analysis of the crude BS carried out in our previous work indicated the presence of polysaccharide, peptide, and lipoic moieties [12]. TLC analysis (Figure S1) showed the presence of amino acid bands corresponding to free amino acids. To this end, before the hydrolysis and the subsequent analysis of the individual amino acids comprising the protein fraction, an extraction of free amino acids was initially carried out. HPLC-DAD analysis verified the bands detected in the TLC plates, as six amino acids were identified in the extract (Table S1).

Seventeen amino acids were identified after the hydrolysis of the protein fraction of the crude BS (Table 2). Arginine was the main amino acid, corresponding to a content of 20.8 mg/g of BS, followed by proline (15.1 mg/g) and glutamic acid (12.2 mg/g BS). As in the case of the individual fatty acids comprising the lipoic part of the molecule, there is a huge variation also in terms of the composition of the peptide part, customized depending on the strain and culture conditions. The amino acid composition of the peptide moiety of BS molecules has not been investigated in LAB-derived BS. Valine, leucine, glutamate, and aspartate have been identified in surfactin [54]. The lipopeptide-type BS produced by *Pseudomonas antarctica* 28E was composed of different amino acids, including leucine, threonine, valine, serine, and isoleucine [55]. In another study, nine amino acids were determined as part of the BS of a lipopeptide synthesized by another *Pseudomonas* strain, with alanine comprising the main component [56]. Clearly, the type and the number of the amino acids present a wide diversity.

#### 3.3.3. Analysis of the Carbohydrate Moiety

HPLC analysis using the RI detector and the column ROA-Rezex revealed 3 peaks after the hydrolysis of BS using TFA and high temperature (Figure S2A). The main peaks corresponded to glucose (71 mg/g BS) and galactose (22.3 mg/g BS), as confirmed using spiking of each compound (Figure S2B,C). The third peak eluting at approximately 15 min was identified as glycerol at a concentration of 14.6 mg/g BS. As already stated, there is great heterogeneity regarding specific BS composition, and the carbohydrate moiety follows the same pattern.

### 3.4. Fractionation of BS Produced from L. fermentum ACA-DC 0183

The capability of lactobacilli to produce a plethora of compounds of different natures (glycoproteins, glycolipoproteins, lipopeptides, glycolipids, etc.) has been highlighted by many researchers [16,26]. Consequently, column fractionation was carried out, aiming to separate the different components of the crude BS.

For the 1st trial, a mixture of chloroform and methanol at various concentrations was selected, since it is the most common solvent system employed in column chromatography for BS fractionation [26,27,28]. Glycolipid-type BS produced by LAB strains has been previously fractionated with gradient mixtures of chloroform: methanol prior to TLC analysis [4,26,57]. However, the BS sample presented poor solubility to these solvent mixtures, creating emulsions in the column. These emulsified zones caused irregular flow, rendering distinct separation challenging. Although different fractions were collected, most retained surface activity, suggesting co-elution of structurally related compounds. The lowest ST values (about 30 mN/m) were attained when the elution was carried out with 100% methanol. Characteristic ATR-FTIR chromatograms are presented in the Supplementary Materials (Figure S3). However, due to the solubility issues, a second trial followed, using a different solvent system.

**Figure 3:**
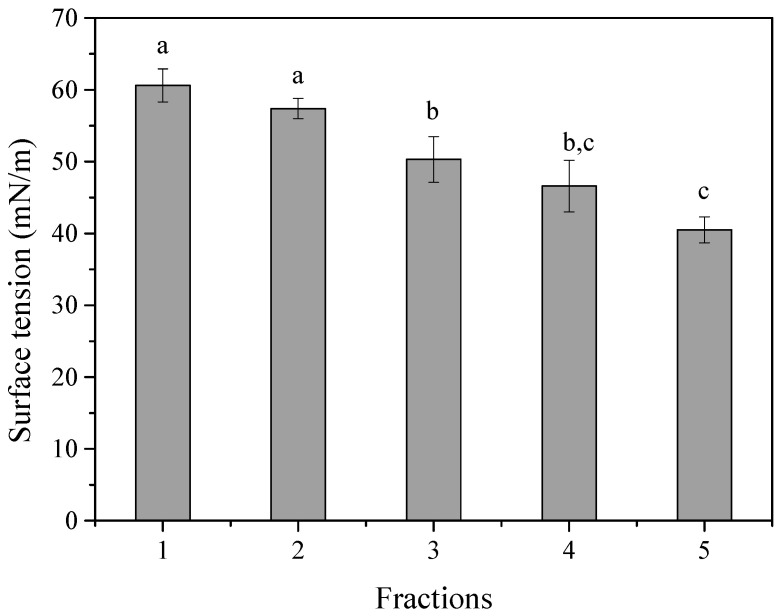
Effect on surface tension of the fractions F1–F5 obtained by a gradient system of acetonitrile: ddH_2_O at concentrations of 0.7 g/L. Results are presented as mean ± standard deviation from triplicate experiments of surface tension measurements. Different lowercase letters present significant differences (*p* < 0.05) among the variables.

For the 2nd trial, a solvent system of acetonitrile and ddH_2_O was evaluated, and 5 fractions were collected. The first fraction (F1) contained the 64.6% of the total BS added in the column, eluted with a mixture of acetonitrile: ddH_2_O (5:95). In the second fraction (F2), the 31.9% of the total mass was extracted while the other three fractions (F3, F4 and F5) contained 1.2, 0.99 and 1.3% of the total added mass, respectively. A solution containing 0.7 g/L of each fraction was prepared, and the surface tension (ST) was measured. As shown in Figure 3, all fractions reduced ST by more than 10 mN/m, indicating the presence of biosurfactants. F4 and F5 resulted in the lowest ST values of 46.6 and 40.5 mN/m, respectively (Figure 3). It is evident that the strain produces a complex BS mixture, in which the most surface-active components are present in relatively lower concentrations. It could be postulated that the strain produces the most highly active molecules in lower amounts. Future research should focus either on identifying the culture conditions promoting the synthesis of these compounds or on their selective recovery.

**Figure 4:**
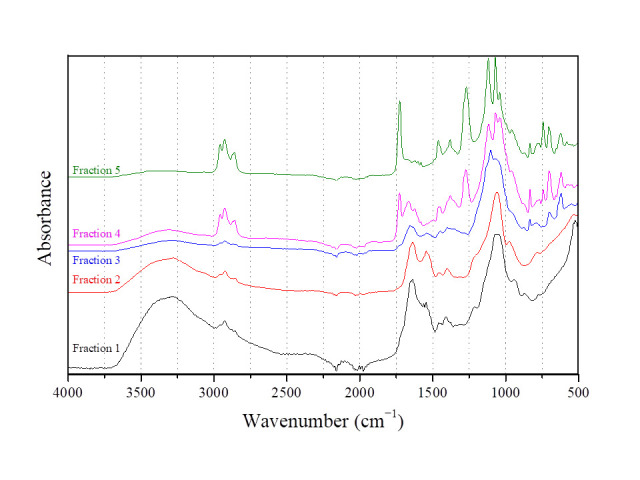
Comparative representation of the IR spectra of the ATR-FTIR analysis of the five fractions.

Aiming to better clarify the nature of the BS derived by *L. fermentum* ACA-DC 0183, the five fractions separated by column chromatography were further analyzed by ATR-FTIR. Figure 4 presents a comparison of the spectra of the five fractions, indicating the separation of different groups of compounds. More specifically, total fractions could be distinguished into 2 groups: F1–F3 and F4 and F5. The first three fractions present similar absorbance, while F2 exhibited the highest purity.

**Figure 5:**
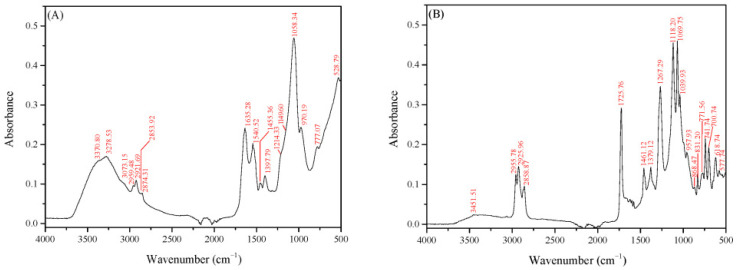
IR spectra of (**A**) fraction 2 (F2) and (**B**) fraction 5 (F5).

In Figure 5, the IR spectra of F2 (Figure 5A) and F5 (Figure 5B) are illustrated, along with the wavenumber of the most characteristic absorbance peaks. Analyzing the main peaks in the case of F2, it can be concluded that the BS separated in this fraction are mainly of a proteinaceous nature, along with a carbohydrate moiety. More specifically, the absorbance with the high intensity in the region 3500–3000 cm^−1^ is the result of three characteristic bands. The bands at 3278.53 cm^−1^ and 3073.15 cm^−1^ are indicative of the presence of proteins: the Amide A band at 3278.53 cm^−1^, which is the result of NH stretching vibration (3310–3270 cm^−1^), and Amide B at 3073.15 cm^−1^ (3100–3030 cm^−1^) due to Fermi resonance phenomenon with Amide A [58]. Additionally, two more characteristic bands of protein structure are observed in the F2 spectrum: the Amide I band with absorbance near 1650 cm^−1^ (at 1635.28 cm^−1^), which is mainly attributed to the C = O stretching vibration of the peptide bond, and the Amide II near 1550 cm^−1^ (at 1540.52 cm^−1^) due to the NH in-plane bending along with CN stretching vibration [59].

The presence of carbohydrates in the molecule could be explained by the third broad band in the same region. This broad band is associated with the O-H stretching vibration, while the resulting broad peak is due to the various types of hydrogen bonds in carbohydrates [59]. Additionally, the bands in 3000–2800 cm^−1^ can be assigned to the antisymmetric stretching vibration of CH_3_ at 2959.48 cm^−1^ and CH_2_ at 2921.69 cm^−1^, as well as the symmetric stretching vibration of these groups at 2874.31 and 2853.92 cm^−1,^ respectively. Moreover, the intense absorbance near 1100 cm^−1^ (at 1060 cm^−1^) is due to the C-O stretching vibration of the C-O-C glycosidic linkage of carbohydrates (ether bond) [60].

Contrary to fractions 1–3, in F4 and F5, the characteristic bands of proteins are not observed. Both fractions present similar spectra, with F5 exhibiting the highest purity. Based on the spectrum of F5 (Figure 5B), several characteristic bands of lipids and carbohydrates are monitored. In particular, the bands in the region 3000–2800 cm^−1^ can be assigned to symmetric and antisymmetric CH_3_ and CH_2_ stretching vibrations. The intensity of these bands is stronger compared to the ones in F2 due to the presence of fatty acids. Since HPLC analysis revealed glycerol as a component of the crude BS, it can be deduced that the absorbance at 1725 cm^−1^ and 1267 cm^−1^ can be correlated to C = O and C-O stretching vibrations of the ester bond between glycerol and fatty acids [60]. Moreover, the bands at 1461 and 1379 cm^−1^ are the result of CH_2_-OH bending and CH_2_ or CH wagging combination [59]. The peaks in the region 1150–1000 cm^−1^ are representative of C-C-O out-of-phase stretching of primary (1075–1000 cm^−1^) and secondary alcohols (1150–1075 cm^−1^), and out-of-phase C-O-C stretching (1150–1060 cm^−1^) of the ether bond between carbohydrates and fatty acids [60]. Characteristic bands of ether bonds can also be observed at 890–820 cm^−1^ because of the in-phase C-O-C stretching vibration. On the other hand, bands at 771.56 and 741.54 cm^−1^ are indicative of skeleton bending of the pyranose ring [61], explaining the presence of glucose and galactose in the structure. Based on the spectrum of F5 and taking into account the HPLC analysis of the hydrolyzed crude sample, a possible explanation is the formation of a glycolipid similar to that once reported by Sauvageau et al. [23] for the biosurfactants produced by *L. plantarum*.

Overall, the IR spectra of fractions 1–5 indicate that the strain is able to produce different compounds exhibiting surfactant properties. The two main structures identified are those of a glycoprotein (fractions 1–3) and of a glycolipid (fractions 4–5). The proteins or lipids are probably connected to glucose or galactose or to both sugars. The glycolipid has a similar structure to the glycolipids produced by *L. plantarum* [23], with a glycerol molecule as a connective part between the carbohydrate and the lipid.

## 4. Conclusions

In this study, crude BS produced by the LAB strain *Limosilactobacillus fermentum* ACA-DC 0183 was characterized for specific functional properties, and its individual components were analyzed. The produced BS was quite resistant to various temperature, pH, and salinity conditions, increasing their potential applications, especially in the food industry. Structural analysis revealed the main components comprising the lipoic, peptide, and carbohydrate moieties of the molecules. The partial fractionation using column chromatography demonstrated the presence of molecules of glycopeptide and glycolipid nature, signifying the ability of LAB strains to produce diversified molecules with surface-active properties. Future studies should therefore focus initially on the fermentation process to unravel when and under which conditions the production of each molecule occurs, along with possible compositional variations depending on the fermentation time and growth state. Further elucidation of the molecular linkages will benefit from more advanced analytical approaches such as LC/GC-MS and/or NMR. Nonetheless, the present work already approaches to a substantial extent the overall molecular composition and points to the coexistence of two structurally distinct BS types, an observation of particular interest given the inherent structural complexity typically associated with LAB-derived BS.

## Figures and Tables

**Table 1:** Fatty acid analysis of the lipoic fraction of crude BS derived from *L. fermentum* ACA-DC 0183.

Composition	Retention Time (min)	Proportion (%)
caproic acid (C6:0)	6.592	0.10
caprylic acid (C8:0)	13.690	0.34
decanoic acid (C10:0)	22.721	2.22
lauric acid (C12:0)	31.596	1.38
myristic acid (C14:0)	39.745	4.25
pentadecanoic acid (C15:0)	43.539	0.46
**palmitic acid (C16:0)**	47.191	**27.89**
palmitoleic acid (C16:1)	48.963	1.23
**stearic acid (C18:0)**	53.968	**11.19**
**oleic acid (C18:1-** ***cis*** ** (n9))**	55.356	**18.67**
linoleic acid (C18:2-*cis* (n6))	57.851	1.34
arachidic acid (C20:0)	60.204	0.27
α-linolenic acid (C18:3 (*ω*-3))	60.808	0.28
eicosenoic acid (C20:1)	61.453	0.37
henicosanoic acid (C21:0)	63.091	1.04
eicosadienoic acid (C20:2)	63.678	0.55
docosanoic acid (C22:0)	66.016	0.16
eicosapentenoic acid (C20:5)	71.591	0.35
tetracosanoic acid (C24:0)	72.581	0.27
docosahexenoic acid (C22:6)	78.523	0.50

**Table 2:** Analysis of amino acid composition obtained after hydrolysis of the protein fraction of crude BS from *L. fermentum* ACA-DC 0183.

Amino Acid	mg/g BS
L-Aspartic acid	6.12 ± 0.11
**L-Glutamic acid**	**12.25 ± 0.32**
L-Serine	3.06 ± 0.05
L-Histidine	9.26 ± 0.26
Glycine	2.11 ± 0.01
L-Threonine	3.72 ± 0.19
**L-Arginine**	**20.83 ± 0.95**
L-Alanine	1.75 ± 0.03
L-Tyrosine	2.85 ± 0.11
L-Cystine	6.80 ± 0.04
L-Valine	4.46 ±0.02
L-Methionine	2.87 ± 0.04
L-Phenylalanine	3.57 ± 0.12
L-Isoleucine	3.32 ± 0.07
L-Leucine	5.13 ± 0.06
L-Lysine	6.45 ± 0.02
**L-Proline**	**15.14 ± 0.22**

## Data Availability

The original contributions presented in this study are included in the article/Supplementary Materials. Further inquiries can be directed to the corresponding author.

## Supplementary Materials

The following supporting information can be downloaded at: https://www.mdpi.com/article/10.3390/foods14244342/s1, Table S1: Analysis of free amino acids contained in the crude BS from *L. fermentum* ACA-DC 0183. Figure S1: TLC plates of crude biosurfactants derived from ACA-DC 0183. Figure S2. HPLC-RID chromatograms of monosaccharide analysis (1 glucose; 2 galactose; 3 glycerol) of the crude BS after hydrolysis with TFA. Figure S3. Characteristic IR spectra of the ATR-FTIR analysis of the fractions obtained during column fraction with chloroform: methanol (Trial 1).

## Abbreviations

The following abbreviations are used in this manuscript:

LAB	Lactic acid bacteria
BS	Biosurfactants
CB-BS	Cell-bound biosurfactants
CW	Cheese whey
DAD	Diode Array Detector
FAN	Free amino nitrogen
FID	Flame Ionization Detector
FTIR	Fourier-Transform Infrared Spectroscopy
GC	Gas chromatography
MRS	De Man, Rogosa, and Sharpe
RP-HPLC	Reverse-phase High-Performance Liquid Chromatography
ST	Surface tension
TDW	Total dry weight
TLC	Thin-Layer Chromatography

## References

[1] da Silva P.F.F., da Silva R.R., Sarubbo L.A., Guerra J.M.C. (2024). Production and optimization of biosurfactant properties using *Candida mogii* and Licuri oil (*Syagrus coronata*). Foods.

[2] Lara V.M., Mendonça C.M.N., Silva F.V.S., Marguet E.R., Vallejo M., Converti A., Varani A.M., Gliemmo M.F., Campos C.A., Oliveira R.P.S. (2022). Characterization of *Lactiplantibacillus plantarum* Tw226 strain and its use for the production of a new membrane-bound biosurfactant. J. Mol. Liq..

[3] Banat I.M., Franzetti A., Gandolfi I., Bestetti G., Martinotti M.G., Fracchia L., Smyth T.J., Marchant R. (2010). Microbial biosurfactants production, applications and future potential. Appl. Microbiol. Biotechnol..

[4] Thavasi R., Jayalakshmi S., Banat I.M. (2011). Application of biosurfactant produced from peanut oil cake by *Lactobacillus delbrueckii* in biodegradation of crude oil. Bioresour. Technol..

[5] Biselli A., Willenbrink A.L., Leipnitz M., Jupke A. (2020). Development, evaluation, and optimisation of downstream process concepts for rhamnolipids and 3-(3-hydroxyalkanoyloxy)alkanoic acids. Sep. Purif. Technol..

[6] Pinto M.I.S., Guerra J.M.C., Meira H.M., Sarubbo L.A., de Luna J.M. (2022). A biosurfactant from *Candida bombicola*: Its synthesis, characterization, and its application as a food emulsions. Foods.

[7] Sittisart P., Gasaluck P. (2022). Biosurfactant production by *Lactobacillus plantarum* MGL-8 from mango waste. J. Appl. Microbiol..

[8] Sharma D., Saharan B.S., Kapil S. (2016). Biosurfactants of probiotic lactic acid bacteria. Biosurfactants of Lactic Acid Bacteria.

[9] Ribeiro B.G., Guerra J.M.C., Sarubbo L.A. (2020). Biosurfactants: Production and application prospects in the food industry. Biotechnol. Prog..

[10] Behzadnia A., Moosavi-nasab M., Tiwari B.K., Setoodeh P. (2020). *Lactobacillus plantarum*- derived biosurfactant: Ultrasound-induced production and characterization. Ultrason. Sonochem..

[11] Lara V.M., Gliemmo M.F., Vallejo M., González M.d.C.G., Rodríguez M.d.C.A., Campos C.A. (2025). Use of the Glycolipopeptid Biosurfactant Produced by *Lactiplantibacillus plantarum* Tw226 to Formulate Functional Cinnamon Bark Essential Oil Emulsions. Foods.

[12] Kachrimanidou V., Alimpoumpa D., Papadaki A., Lappa I., Alexopoulos K., Kopsahelis N. (2022). Cheese whey utilization for biosurfactant production: Evaluation of bioprocessing strategies using novel Lactobacillus strains. Biomass Convers. Biorefinery.

[13] Kachrimanidou V., Papadaki A., Lappa I., Papastergiou S., Kleisiari D., Kopsahelis N. (2022). Biosurfactant production from Lactobacilli: An insight on the interpretation of prevailing assessment methods. Appl. Biochem. Biotechnol..

[14] Kachrimanidou V., Alexandri M., Nascimento M.F., Alimpoumpa D., Torres Faria N., Papadaki A., Castelo Ferreira F., Kopsahelis N. (2022). Lactobacilli and *Moesziomyces* biosurfactants: Toward a closed-loop approach for the dairy industry. Fermentation.

[15] Vučurović D., Bajić B., Trivunović Z., Dodić J., Zeljko M., Jevtić-Mučibabić R., Dodić S. (2024). Biotechnological Utilization of Agro-Industrial Residues and By-Products—Sustainable Production of Biosurfactants. Foods.

[16] Mouafo H.T., Sokamte A.T., Mbawala A., Ndjouenkeu R., Devappa S. (2022). Biosurfactants from lactic acid bacteria: A critical review on production, extraction, structural characterization and food application. Food Biosci..

[17] Mouafo H.T., Mbawala A., Somashekar D., Tchougang H.M., Harohally N.V., Ndjouenkeu R. (2021). Biological properties and structural characterization of a novel rhamnolipid like-biosurfactants produced by *Lactobacillus casei* subsp. *casei*
*TM1B*. Biotechnol. Appl. Biochem..

[18] Venkataraman S., Rajendran D.S., Kumar P.S., Vo D.V.N., Vaidyanathan V.K. (2022). Extraction, purification and applications of biosurfactants based on microbial-derived glycolipids and lipopeptides: A review. Environ. Chem. Lett..

[19] Kachrimanidou V., Alexandri M., Alimpoumpa D., Papadaki A., Lappa I.K., Kopsahelis N. (2023). Biosurfactants production by LAB and emerging applications. Lactic Acid Bacteria as Cell Factories.

[20] Mouafo H.T., Sokamte A.T., Manet L., Mbarga A.J.M., Nadezdha S., Devappa S., Mbawala A. (2023). Biofilm inhibition, antibacterial and antiadhesive properties of a novel biosurfactant from *Lactobacillus paracasei* N2 against multi-antibiotics-resistant pathogens isolated from braised fish. Fermentation.

[21] Nataraj B.H., Ramesh C., Mallappa R.H. (2021). Characterization of biosurfactants derived from probiotic lactic acid bacteria against methicillin-resistant and sensitive Staphylococcus aureus isolates. Lwt.

[22] Saravanakumari P., Mani K. (2010). Structural characterization of a novel xylolipid biosurfactant from *Lactococcus lactis* and analysis of antibacterial activity against multi-drug resistant pathogens. Bioresour. Technol..

[23] Sauvageau J., Ryan J., Lagutin K., Sims I.M., Stocker B.L., Timmer M.S.M. (2012). Isolation and structural characterisation of the major glycolipids from *Lactobacillus plantarum*. Carbohydr. Res..

[24] Murphy B.F., Mulvihill D.M. (1988). Proteins recovered from acid whey/skim milk mixtures heated at alkaline pH values. Int. J. Dairy Technol..

[25] Grufferty M.B., Mulvihill D.M. (1987). Proteins recovered from milks heated at alkaline pH values. Int. J. Dairy Technol..

[26] Sharma D., Saharan B.S., Chauhan N., Procha S., Lal S. (2015). Isolation and functional characterization of novel biosurfactant produced by *Enterococcus faecium*. Springerplus.

[27] Gogoi D., Bhagowati P., Gogoi P., Bordoloi N.K., Rafay A., Dolui S.K., Mukherjee A.K. (2016). Structural and physico-chemical characterization of a dirhamnolipid biosurfactant purified from: *Pseudomonas aeruginosa*: Application of crude biosurfactant in enhanced oil recovery. RSC Adv..

[28] Zargar A.N., Lymperatou A., Skiadas I., Kumar M., Srivastava P. (2022). Structural and functional characterization of a novel biosurfactant from *Bacillus* sp. IITD106. J. Hazard. Mater..

[29] Mouafo T.H., Mbawala A., Ndjouenkeu R. (2018). Effect of different carbon sources on biosurfactants’ production by three strains of *Lactobacillus* spp.. BioMed Res. Int..

[30] Sakr E.A.E., Ahmed H.A.E., Abo Saif F.A.A. (2021). Characterization of low-cost glycolipoprotein biosurfactant produced by *Lactobacillus plantarum* 60 FHE isolated from cheese samples using food wastes through response surface methodology and its potential as antimicrobial, antiviral, and anticancer activi. Int. J. Biol. Macromol..

[31] Lie S. (1973). The EBC-ninhydrin method for determination of free alpha amino nitrogen. J. Inst. Brew..

[32] Lowry O.H., Rosebrough N.J., Farr A.L., Randall R.J. (1951). Protein measurement with the Folin phenol reagent. J. Biol. Chem..

[33] Dubois M., Gilles K.A., Hamilton J.K., Rebers P.A., Smith F. (1956). Colorimetric method for determination of sugars and related substances. Anal. Chem..

[34] Patel A., Pruthi V., Singh R.P., Pruthi P.A. (2015). Synergistic effect of fermentable and non-fermentable carbon sources enhances TAG accumulation in oleaginous yeast *Rhodosporidium kratochvilovae* HIMPA1. Bioresour. Technol..

[35] Kowalska S., Szłyk E., Jastrzębska A. (2022). Simple extraction procedure for free amino acids determination in selected gluten-free flour samples. Eur. Food Res. Technol..

[36] Shen P., Gao Z., Xu M., Ohm J.B., Rao J., Chen B. (2020). The impact of hempseed dehulling on chemical composition, structure properties and aromatic profile of hemp protein isolate. Food Hydrocoll..

[37] Natsia A., Papadaki A., Papapostolou H., Christopoulou N.M., Kopsahelis N. (2025). Effect of solubilization pH and dehulling on hempseed protein recovery: Chemical characterization, physical properties, and preliminary study of carbon dots synthesis from residual solids. Sustain. Chem. Pharm..

[38] Olszewska-Widdrat A., Babor M., Höhne M.M.C., Alexandri M., López-Gómez J.P., Venus J. (2024). A mathematical model-based evaluation of yeast extract’s effects on microbial growth and substrate consumption for lactic acid production by *Bacillus coagulans*. Process Biochem..

[39] Klotz S., Kuenz A., Prüße U. (2017). Nutritional requirements and the impact of yeast extract on the d-lactic acid production by Sporolactobacillus inulinus. Green Chem..

[40] Meng L., Li S., Liu G., Fan X., Qiao Y., Zhang A., Lin Y., Zhao X., Huang K. (2021). The nutrient requirements of *Lactobacillus acidophilus* LA-5 and their application to fermented milk. J. Dairy Sci..

[41] Yeboah P.J., Ibrahim S.A., Krastanov A. (2023). A review of fermentation and the nutritional requirements for effective growth media for lactic acid bacteria. Food Sci. Appl. Biotechnol..

[42] Hsieh C.M., Yang F., Iannotti E.L. (1999). The effect of soy protein hydrolyzates on fermentation by *Lactobacillus amylovorus*. Process Biochem..

[43] Vallejo M.C., Restrepo M.A.F., Duque F.L.G., Díaz J.C.Q. (2021). Production, characterization and kinetic model of biosurfactant produced by lactic acid bacteria. Electron. J. Biotechnol..

[44] Souza E.C., Oliveira de Souza de Azevedo P., Domínguez J.M., Converti A., Pinheiro De Souza Oliveira R. (2017). Influence of temperature and pH on the production of biosurfactant, bacteriocin and lactic acid by *Lactococcus lactis* CECT-4434. CyTA J. Food.

[45] Ghasemi A., Moosavi-Nasab M., Setoodeh P., Mesbahi G., Yousefi G. (2019). Biosurfactant production by lactic acid bacterium *Pediococcus dextrinicus* SHU1593 grown on different carbon sources: Strain screening followed by product characterization. Sci. Rep..

[46] Gudiña E.J., Rodrigues A.I., Alves E., Domingues M.R., Teixeira J.A., Rodrigues L.R. (2015). Bioconversion of agro-industrial by-products in rhamnolipids toward applications in enhanced oil recovery and bioremediation. Bioresour. Technol..

[47] El-Housseiny G.S., Aboshanab K.M., Aboulwafa M.M., Hassouna N.A. (2020). Structural and physicochemical characterization of rhamnolipids produced by *Pseudomonas aeruginosa* P6. AMB Express.

[48] Mouafo H.T., Mbawala A., Tanaji K., Somashekar D., Ndjouenkeu R. (2020). Improvement of the shelf life of raw ground goat meat by using biosurfactants produced by lactobacilli strains as biopreservatives. Lwt.

[49] Vecino X., Rodríguez-López L., Gudiña E.J., Cruz J.M., Moldes A.B., Rodrigues L.R. (2017). Vineyard pruning waste as an alternative carbon source to produce novel biosurfactants by *Lactobacillus paracasei*. J. Ind. Eng. Chem..

[50] Thakur B., Kaur S., Dwibedi V., Albadrani G.M., Al-Ghadi M.Q., Abdel-Daim M.M. (2024). Unveiling the antimicrobial and antibiofilm potential of biosurfactant produced by newly isolated *Lactiplantibacillus plantarum* strain 1625. Front. Microbiol..

[51] Al-janabi M.Z.J., Al-dulaimy W.Y.M., Abdulateef M.H., Ahmed A.A., Kadhom M. (2025). Comparative cytotoxicity of a glycolipopeptide biosurfactant from *Lactobacillus plantarum* and its derived silver nanoparticles against breast cancer cells. Med. Microecol..

[52] Roy A., Khan M.R., Mukherjee A.K. (2025). Statistical optimization of production and characterization of biosurfactant produced by *Lactobacillus plantarum* JBC5 strain in a ghee (clarified butter) medium: Assessment of its antimicrobial activity and stain removal property. Process Biochem..

[53] de Andrade C.J., de Andrade L.M., Rocco S.A., Sforça M.L., Pastore G.M., Jauregi P. (2017). A novel approach for the production and purification of mannosylerythritol lipids (MEL) by *Pseudozyma tsukubaensis* using cassava wastewater as substrate. Sep. Purif. Technol..

[54] Kumari R., Singha L.P., Shukla P. (2023). Biotechnological potential of microbial bio-surfactants, their significance, and diverse applications. FEMS Microbes.

[55] Ciurko D., Chebbi A., Kruszelnicki M., Czapor-Irzabek H., Urbanek A.K., Polowczyk I., Franzetti A., Janek T. (2023). Production and characterization of lipopeptide biosurfactant from a new strain of *Pseudomonas antarctica* 28E using crude glycerol as a carbon source. RSC Adv..

[56] Wang G., Wang Y., Chen Y., Ma F. (2025). Lipopeptide biosurfactants of Pseudomonas fragi showed intraspecific specificity to their biological traits. Food Biosci..

[57] Sharma D., Saharan B.S., Chauhan N., Bansal A., Procha S. (2014). Production and structural characterization of *Lactobacillus helveticus* derived biosurfactant. Sci. World J..

[58] Barth A. (2007). Infrared spectroscopy of proteins. Biochim. Biophys. Acta.

[59] Lin-Vien D., Colthup N.B., Fateley W.G., Grasselli J.G. (1991). The Handbook of Infrared and Raman Characteristic Frequencies of Organic Molecules.

[60] Stuart B.H. (2004). Infrared Spectroscopy: Fundamentals and Applications.

[61] Barbosa-Pereira L., Pocheville A., Angulo I., Paseiro-Losada P., Cruz J.M. (2013). Fractionation and purification of bioactive compounds obtained from a brewery waste stream. BioMed Res. Int..

