# Supplementary material for: Fractionation and Chemical Characterization of Cell-Bound Biosurfactants Produced by a Novel Limosilactobacillus fermentum Strain via Cheese Whey Valorization
Source: Foods. 2025 Dec 17;14(24):4342. doi: 10.3390/foods14244342 (PMC12732783; doi:10.3390/foods14244342)
Supplement: Supplementary file 1 [file foods-14-04342-s001.zip › foods-3981584-supplementary.pdf]

# Supplementary Material

# Fractionation and Chemical Characterization of Cell-Bound Biosurfactants Produced by a Novel *Limosilactobacillus fermentum* Strain via Cheese Whey Valorisation

**Dimitra Alimpoumpa, Harris Papapostolou, Maria Alexandri, Vasiliki Kachrimanidou, Nikolaos Kopsahelis***

*Department of Food Science and Technology, Ionian University, Argostoli 28100, Kefalonia, Greece* ; dimialib2014@yahoo.com; harris_papapostolou@yahoo.gr; malexandri@ionio.gr; v.kachrimanidou@ionio.gr

* Correspondence Dr. N. Kopsahelis, Department of Food Science & Technology, Ionian University, Argostoli, 28100, Kefalonia, Greece. E-mail: kopsahelis@ionio.gr, kopsahelis@upatras.gr, phone number: +30 26710 26505)

**Table S1:** Analysis of free amino acids contained in the crude BS from *L. fermentum* ACA-DC 0183.

| Amino acid | mg/g BS |
|---|---|
| L-Glutamic acid | 0.41 ± 0.01 |
| L-Histidine | 0.53 ± 0.03 |
| Glycine | 0.21 ± 0.01 |
| L-Arginine | 2.68 ± 0.11 |
| L-Tyrosine | 0.14 ± 0.01 |
| L-Valine | 0.20 ± 0.01 |

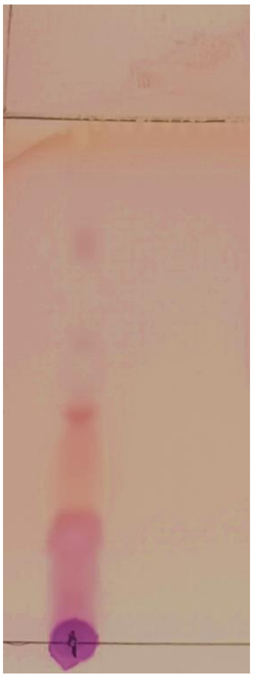

**ACA-DC 183**

**Figure S1**: TLC plates of crude biosurfactants derived from ACA-DC 0183. The plates were developed using the solvent system $CHCl_3$: $CH_3OH$: $CH_3COOH$: $dH_2O$ and stained with a ninhydrin solution in ethanol.

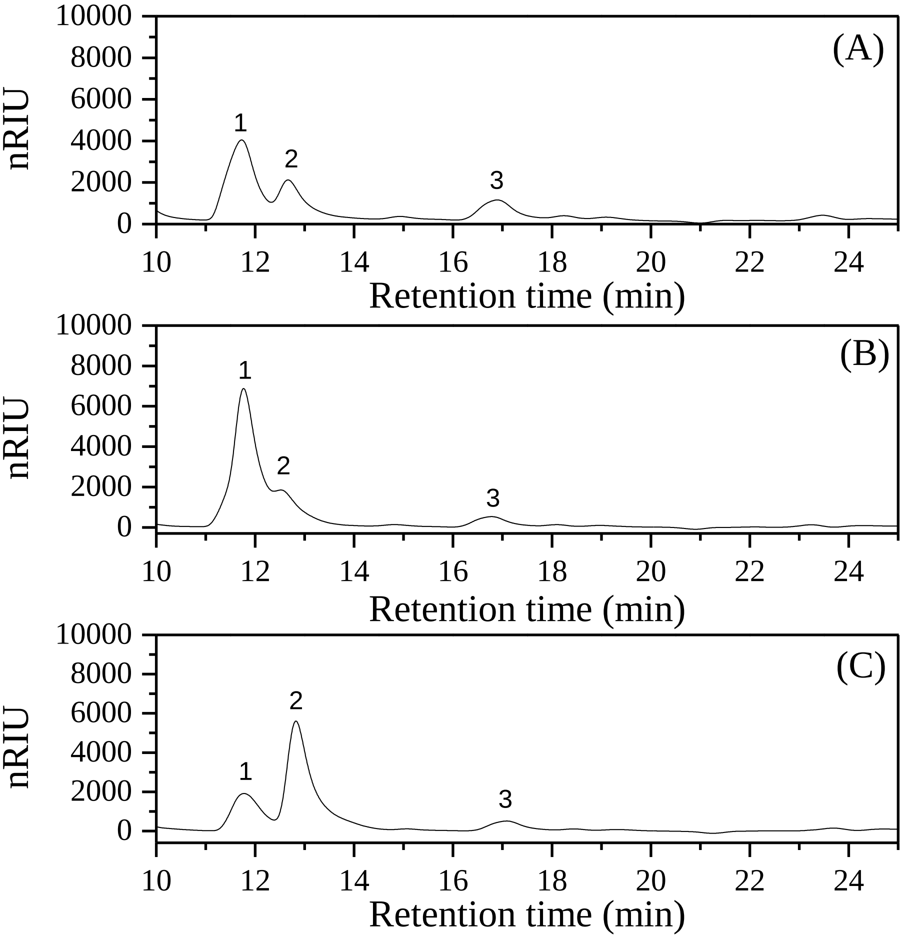


**Figure S2.** HPLC-RID chromatograms of monosaccharide analysis (1 glucose; 2 galactose; 3 glycerol) of the crude BS after hydrolysis with TFA. (A) crude BS after hydrolysis, (B) crude BS sample after hydrolysis after addition of glucose (spiking), (C) crude BS sample after hydrolysis after addition of galactose (spiking).

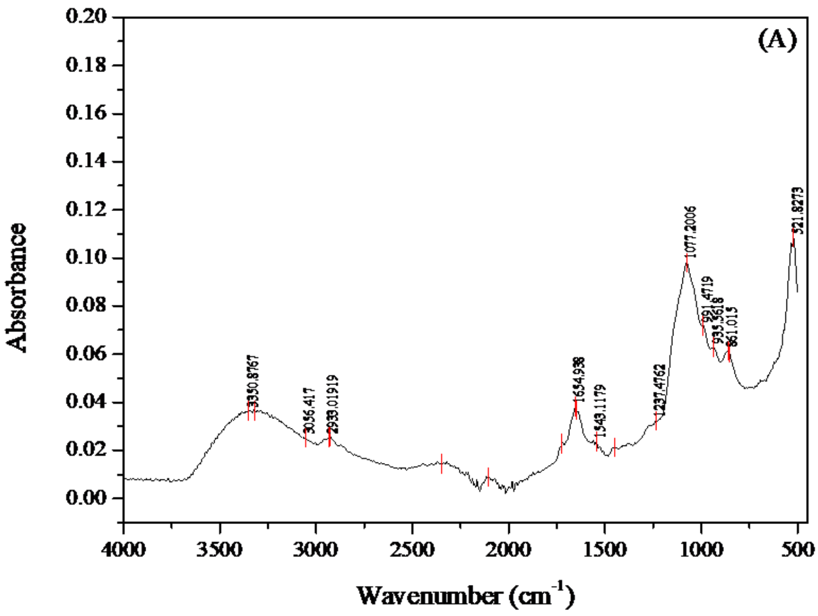


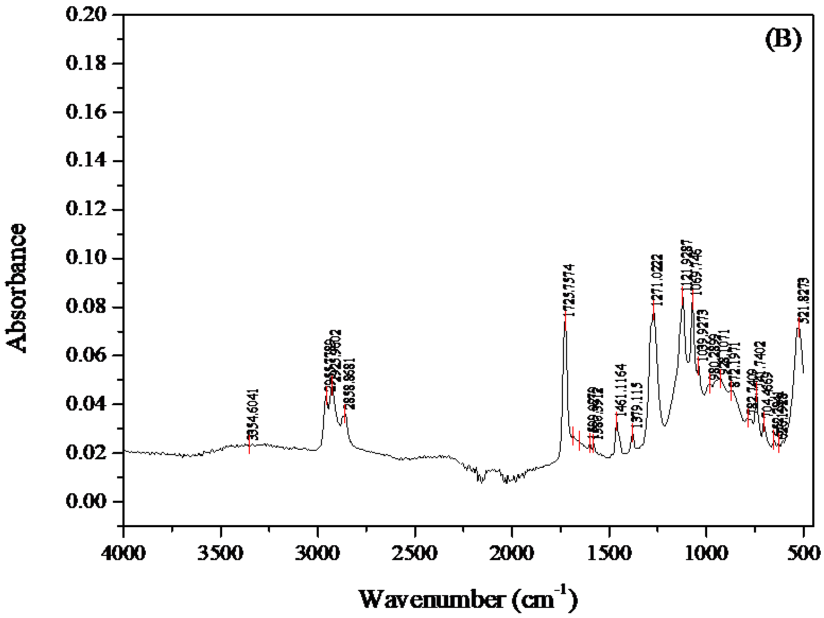


**Figure S3.** Characteristic IR spectra of the ATR-FTIR analysis of the fractions obtained during column fraction with chloroform: methanol (Trial 1).
